
JOURNAL INFORMATION
==============================
NLM Title Abbreviation: Wirtschaftsdienst
Journal ID: Wirtschaftsdienst
NLM Journal ID: 101086612

Wirtschaftsdienst (Hamburg, Germany : 1949)

ISSN: 0043-6275

ARTICLE INFORMATION
==============================
PMCID: PMC7174813
PMID: 32336799
DOI: 10.1007/s10273-020-2626-2
Article ID: 2626
Article version: 1
Subjects: Zeitgespräch

Vermögensverteilung und Wirtschaftskrisen

Rehm Miriam miriam.rehm@uni-due.de

grid.5718.b 0000 0001 2187 5445 Institut für Sozioökonomie - Gesellschaftswissens, Universität Duisburg-Essen, Lotharstraße 65, 47057 Duisburg, Deutschland
Electronic publication date: 2020 Apr 22
Print publication date: 2020
Volume: 100
Issue: 4
First page: 245
Last page: 249
Copyright: © Der/die Autor(en) 2020
License: Open Access Dieser Artikel wird unter der Creative Commons Namensnennung 4.0 International Lizenz veröffentlicht, welche die Nutzung, Vervielfältigung, Bearbeitung, Verbreitung und Wiedergabe in jeglichem Medium und Format erlaubt, sofern Sie den/die ursprünglichen Autor(en) und die Quelle ordnungsgemäß nennen, einen Link zur Creative Commons Lizenz beifügen und angeben, ob Änderungen vorgenommen wurden.
Die in diesem Artikel enthaltenen Bilder und sonstiges Drittmaterial unterliegen ebenfalls der genannten Creative Commons Lizenz, sofern sich aus der Abbildungslegende nichts anderes ergibt. Sofern das betreffende Material nicht unter der genannten Creative Commons Lizenz steht und die betreffende Handlung nicht nach gesetzlichen Vorschriften erlaubt ist, ist für die oben aufgeführten Weiterverwendungen des Materials die Einwilligung des jeweiligen Rechteinhabers einzuholen.
Weitere Details zur Lizenz entnehmen Sie bitte der Lizenzinformation auf http://creativecommons.org/licenses/by/4.0/deed.de.
License URL: https://creativecommons.org/licenses/by/4.0/

issue-copyright-statement: © The Author(s) 2020

==============================
Prof. Dr. Miriam Rehm ist Juniorprofessorin für Sozioökonomie mit Schwerpunkt Empirische Ungleichheitsforschung am Institut für Sozioökonomie der Universität Duisburg-Essen.

Ökonomische Forschung ebenso wie öffentliche Diskussionen über Verteilung beziehen sich meist auf Einkommen. Das liegt zu einem guten Teil an der Datenverfügbarkeit: Einkommensdaten stehen sowohl in langen historischen Zeitreihen als auch relativ rasch in hoher Qualität und mit Verteilungsdimensionen (und somit nicht nur im Aggregat) aus unterschiedlichen Quellen zur Verfügung. Vermögen ist dagegen in der öffentlichen Debatte häufig unterrepräsentiert, und ist auch deutlich seltener Gegenstand wissenschaftlicher Forschung als Einkommen.

Aber Vermögen hat auch breitere, sozioökonomische Aspekte. So kann es etwa als Statussymbol dienen und politische Macht verleihen (Fessler und Schürz, 2018). Die Verteilung von privatem Vermögen ist daher aus ökonomischer ebenso wie aus sozialwissenschaftlicher Perspektive ein höchst wichtiges Forschungsthema. Nicht nur, aber insbesondere auch in Krisenzeiten zeigt sich, dass neben dem Privatvermögen zudem das öffentliche Vermögen eine zentrale Rolle für das gesamtgesellschaftliche Wohlergehen darstellt. Dieser Beitrag beschreibt die Vermögensverteilung in Deutschland anhand der Ende März 2020 von der Deutschen Bundesbank erhobenen und der Europäischen Zentralbank publizierten Daten des Household Finance and Consumption Survey (HFCS, in Deutschland PHF, das Panel on Household Finances genannt).[1]

halte abzüglich ihrer Schulden – sind viel ungleicher verteilt als Einkommen (Frick und Grabka, 2007). Der Gini-Koeffizient der Nettovermögen liegt in jedem der 18 europäischen Länder[2], die die neueste Welle des HFCS 2017 abdeckt, deutlich über dem Gini-Koeffizienten der Bruttoeinkommen: Während diese Einkommens-Ginis zwischen 0,35 und 0,54 liegen, liegen die Vermögens-Ginis zwischen 0,55 und 0,8 (Oesterreichische Nationalbank [OeNB], 2020). Deutschland liegt bei der Verteilung der Bruttoeinkommen nach diesen Daten im europäischen Mittelfeld, weist aber eine der höchsten Ungleichverteilungen beim Nettovermögen, dessen Gini-Koeffizient etwa 0,74 ausmacht, auf (Europäische Zentralbank [EZB], 2020).

In absoluten Zahlen macht der Durchschnitt, der für Verzerrungen durch Ausreißer verhältnismäßig anfällig ist, 232.800 Euro aus. Das Medianvermögen – das gegenüber Rauschen in der Datenerhebung robuster ist – liegt bei 70.800 Euro. Die Perzentilsgrenzen liegen in den neuesten Daten für die unteren 25 % der Haushalte bei etwa 6.200 Euro, für die reichsten 25 % bei ca. 268.000 Euro und für die reichsten 5 % der Haushalte bei rund 860.000 Euro.

## Selbsteinschätzung beim Vermögen

Allerdings scheint das Wissen um die Verteilung des Vermögens und die eigene Position in der Vermögensverteilung beschränkt zu sein. Auswertungen der Deutschen Bundesbank (2019) aus den Non-core-Variablen der 2. Welle des HFCS zeigen, dass Haushalte Schwierigkeiten haben, ihre Position richtig einzuschätzen. Interessanterweise – und im Gegensatz zu den Erkenntnissen der Literatur über finanzielle Kompetenzen („financial literacy") – korreliert das Wissen um die eigene Position in der Vermögensverteilung negativ mit der Vermögenshöhe. Insbesondere am oberen Ende der Verteilung ist die Finanzkompetenz in Bezug auf die Vermögensverteilung schwach ausgeprägt, nur 3 % der Haushalte verorten sich in den obersten 30 % der Vermögensverteilung (Deutsche Bundesbank, 2019, 24). Diese Erkenntnisse bestätigen Ergebnisse aus den österreichischen HFCS-Daten, die diese Tendenz in der 1. und 2. Welle ebenfalls feststellen (Fessler et al., 2019). Hier zeigt sich zudem ein negativer Zusammenhang zwischen der Finanzkompetenz in Bezug auf die Vermögensverteilung mit der Einkommenshöhe sowie dem Bildungsgrad (Kranawetter und Rehm, 2018).

2 Belgien, Deutschland, Estland, Griechenland, Irland, Italien, Kroatien, Lettland, Luxemburg, Malta, Niederlande, Österreich, Polen, Portugal, Slowakei, Slowenien, Ungarn und Zypern.

Tabelle 1

**Partizipation an Vermögenskategorien**

Anteil der Haushalte, die Vermögen in dieser Kategorie angelegt haben, in %

| | Untere Hälfte Perzentil 0-50 | Obere Mitte Perzentil 51-80 | Vermögende Perzentil 81-95 | Top 5% Perzentil 96-100 |
|---|---|---|---|---|
| Kraftfahrzeuge | 60 | 88 | 93 | 89 |
| Wertgegenstände | 13 | 18 | 25 | 36 |
| Hauptwohnsitz | 8 | 72 | 90 | 93 |
| weitere Immobilien | 4 | 27 | 56 | 78 |
| Unternehmen | 4 | 12 | 18 | 47 |
| Sichteinlagen | 99 | 100 | 100 | 100 |
| Aktien | 3 | 11 | 27 | 42 |
| Anleihen | 1 | 4 | 7 | 13 |
| Fonds | 6 | 18 | 34 | 41 |
| weiteres Finanzvermögen | 9 | 16 | 27 | 34 |
| Privatpension/ Lebensversicherung | 32 | 52 | 57 | 66 |
| informelle Forderung | 15 | 10 | 10 | 19 |

Quelle: eigene Berechnungen, Daten: HFCS 2017.

## Intuitive Vermögensverteilung

Ein detaillierteres Bild der Verteilung zeichnet die Partizipation unterschiedlicher Vermögensgruppen an den einzelnen Kategorien der Vermögenswerte. Dieses ist nicht nur wenig anfällig für Verzerrungen durch Extremwerte, sondern vermittelt auch ein intuitiveres Verständnis der Vermögensverteilung.

Tabelle 1 teilt deutsche Haushalte nach der Höhe ihres Nettovermögens in vier Gruppen: die untere Hälfte (Perzentil 0 bis 50), die obere Mitte (51 bis 80), die Vermögenden (81 bis 95) und die reichsten 5 %. Beim Sachvermögen ist in der unteren Hälfte der Bevölkerung vor allem der Besitz von Kraftfahrzeugen von Bedeutung, wie Tabelle 1 zeigt. Wohneigentum wird dagegen in der oberen Hälfte relevant, und erst bei den Vermögenden hält mehr als die Hälfte weiteres Immobilieneigentum. Unternehmensbesitz steigt erst bei den reichsten 5 % sprunghaft an. Beim Finanzvermögen sind nur Sichteinlagen (Giro- und Sparkonten) in allen Bevölkerungsgruppen weit verbreitet. Aktien, Investmentfonds, weiteres Finanzvermögen (Indexzertifikate, aber etwa auch komplexere Finanzprodukte) sind ab der Gruppe der „Vermögenden" annähernd in jedem dritten Haushalt Teil des Portfolios. Privatpensionen oder Lebensversicherungen besitzt in der unteren Hälfte beinahe ein Drittel der Haushalte; bei den Top 5 % sind es etwa zwei Drittel. Interessanterweise spielen auch in Deutschland informelle Kredite zwischen Haushalten eine nicht unbedeutende Rolle. Zwischen 10 % und 19 % der Haushalte fallen in diese Vermögenskategorie.

### Vermögen und Geschlecht

Bei Erhebungsdaten zum Vermögen steht dem gravierenden Nachteil der Untererfassung am oberen Ende der Vorteil der detaillierten sozioökonomischen Charakteristika gegenüber. So ist etwa auf Basis der Daten des Sozio-oekonomisches Panels (SOEP) die Vermögensverteilung nach Geschlecht in Deutschland ausnehmend gut erforscht (Sierminska et al., 2010, 2018). Im HFCS zeigen Schneebaum et al. (2018), dass sich die Vermögensschere nach Geschlecht in Deutschland wie in anderen europäischen Ländern vor allem am oberen Ende öffnet: Während im Durchschnitt keine statistisch signifikante Lücke beobachtbar ist, sind am 95. Perzentil die Unterschiede im Bruttovermögen zwischen Ein-Erwachsenen-Haushalten von Frauen und Männern in Deutschland zu 45 % nicht durch Kontrollvariablen[3] erklärbar. Am klarsten zeigen aber die deskriptiven Daten, dass auch bei Vermögen eine „gläserne Decke" herrscht, während über die Breite der Verteilung wenig Unterschiede zwischen Ein-Erwachsenen-Haushalten von Männern und Frauen herrschen.

### Die Rolle von Privatvermögen in Krisenzeiten

Allerdings gibt es sozioökonomische Gruppen, deren Vermögenssituation ihre ökonomische Freiheit in Krisensituationen – wie derzeit aufgrund der COVID-19-Pandemie – deutlich einschränkt. Die Daten aus Tabelle 1 geben erste Hinweise auf mögliche Krisenfolgen für die unterschiedlichen Vermögensgruppen: Der Besitz von Kraftfahrzeugen ermöglicht Mobilität, wenn die Benutzung der öffentlichen Verkehrsmittel aus seuchenhygienischen Gründen eingeschränkt wird. Ein (unbelehntes) Eigenheim zu besitzen, reduziert bei einem kurzfristigen Entgeltausfall die laufenden Ausgaben. Weiteres Immobilienvermögen inkludiert Zweitwohnsitze, die die Flexibilität geben können, in weniger stark betroffene Gebiete auszuweichen oder zumindest Abwechslung in die Wohnsituation zu bringen. Unternehmens- sowie Finanzvermögensbesitz dagegen führen in den meisten Fällen während schwerer Wirtschaftskrisen zu Einkommenseinbußen oder -ausfällen, und sind üblicherweise mit hohen (wenn auch oft nicht realisierten) Wertverlusten konfrontiert.

Grundsätzlich gilt, dass die Sicherheitsfunktion von Vermögen bei sehr geringem Besitz nur ungenügend ausgefüllt werden kann. So besitzen etwa Haushalte, in denen die Referenzperson arbeitslos ist, in Deutschland im HFCS 2017 im Median knapp 600 Euro, die allerdings aufgrund der geringen Fallzahl mit großer Vorsicht zu interpretieren sind. Des Weiteren ist die Nutzungsfunktion von Vermögen für manche Bevölkerungsgruppen deutlich eingeschränkt. So wohnen etwa Alleinerziehende, vornehmlich Frauen, im Durchschnitt auf etwa 34 Quadratmetern pro Person (zu über drei Viertel zur Miete); während die durchschnittliche Wohnfläche in der Gesamtbevölkerung über 56 Quadratmeter pro Person beträgt. Auch hier ist die Fallzahl sehr gering, aber die Varianz deutlich niedriger.

Der Besitz privaten Vermögens spielt somit insbesondere in Krisenzeiten eine wichtige Rolle bei der Abfederung von Risiken. Allerdings muss nicht zwingend Privatvermögen diese Absicherung ausfüllen; auch öffentliches Vermögen ist in der Lage, Schocks abzufedern.

### Die Rolle von öffentlichem Vermögen in Krisenzeiten

Öffentliches Vermögen erfüllt zentrale ökonomische Funktionen in der Bereitstellung öffentlicher Güter (etwa in der Mobilität), in der Sicherung gesamtgesellschaftlichen Wohlstands (etwa bei öffentlichem Wohnbau), und der intertemporalen Allokation (etwa bei Bildung, Gesundheit und Pflege). Diese sind interdependent und ihre Interpretation verändert sich im Zeitverlauf. So wird der Staat in der Ökonomie oft als wenig innovationsfreudig wahrgenommen, obwohl er eine bedeutsame Rolle in der Grundlagenforschung einnimmt (Mazzucato, 2014). Diese Funktion kann auch über Staatsvermögen – etwa in Form von Beteiligungen – vermittelt werden.

Allerdings werden in der öffentlichen Debatte häufig einseitig die Staatsschulden in den Blick genommen, anstatt in einer Nettobetrachtung diesen das öffentliche Vermögen gegenüberzustellen, wie es aus einem ökonomischen Blickwinkel geboten wäre (Organisation für wirtschaftliche Zusammenarbeit und Entwicklung [OECD], 2018, 50; Internationaler Währungsfonds [IWF], 2017). In Krisenzeiten rückt dagegen das öffentliche Bruttovermögen stärker in den Vordergrund. In der Finanz- und Wirtschaftskrise nach 2008 waren dies insbesondere Staatsbeteiligungen an geretteten Finanzin-

3 Diese umfassen Alter, Bildung, Familienstatus, erhaltene Erbschaften, Arbeitsmarktcharakteristika und Vermögenskategorien.

stituten, während in der Gesundheitskrise aufgrund von COVID-19 die Kapazitäten des Gesundheitssystems in den Fokus rücken. Diese reichen von Krankenhäusern über Messegelände für Notbetreuungsstationen, bis zu Katastrophengerät der Bundeswehr.

Die Entwicklung der Krankenhausbetten zur Heilpflege („curative hospital beds"), die in Deutschland zwischen 1991 und 2017 um etwa 25 % gesunken ist (European Commission, 2020), steht dabei beispielhaft für die sinkende Handlungsfähigkeit des Staats in diesem Bereich. In Deutschland sinkt das öffentliche Nettovermögen seit den 1970er Jahren und beträgt seit 2000 etwa null (Piketty, 2014). Das liegt neben den steigenden Staatsschulden an einer schwachen Entwicklung der öffentlichen Investitionen. Seit den späten 1990er Jahren schwanken die öffentlichen Nettoinvestitionen in Deutschland um null; Bardt et al. (2019, 10) schätzen einen rechnerischen Investitionsbedarf in Höhe von 1,3 % der jährlichen Wirtschaftsleistung bis 2030. Trotz aller Bewertungs- und Erhebungsprobleme bei öffentlichen Vermögen gibt das einen Hinweis auf ihren sinkenden relativen Bedeutungsgrad im Verhältnis zu privatem Vermögen.

Dabei spielt insbesondere die Planungskapazität des Staats in der erfolgreichen Erfüllung der Funktionen öffentlichen Vermögens eine wichtige Rolle. Wenn etwa Kommunen geplante Investitionsvorhaben nur schwer durchführen können, zeigt sich darin auch die Schwierigkeit, öffentliches Vermögen klar von Humanressourcen zu trennen. Schließlich gibt es neben der Komplementarität öffentlichen Vermögens mit öffentlichem Personal auch einen Substitutionseffekt – bei ausreichend vorhandener Schutzkleidung können etwa Schichten von Krankenhauspersonal in stark von COVID-19 betroffenen Gebieten verkürzt werden, was wiederum die Ansteckungsgefahr deutlich verringert (Rinke, 2020).

Die derzeitige Krise zeigt zudem auf, dass die Koordination essenzieller Produktion zumindest in Krisenzeiten gesichert sein muss. Die Schwierigkeiten und internationalen Verwerfungen, die die Probleme in der Beschaffung ausreichender Mengen an Schutzkleidung bereiten, führen in mehreren Ländern bereits dazu, dass eine Umstellung der Produktionsketten auf Bedarfsgüter etwa im Textilbereich zentral koordiniert wird. Langfristig wird sich daher die Frage stellen, ob den Funktionen öffentlichen Vermögens nicht eine weitere beigestellt werden muss.

## Vermögensverteilung in der kurzen und langen Frist

Im Gegensatz zur Finanz- und Wirtschaftskrise nach 2008 rücken die Verteilungseffekte der derzeitigen Gesundheits- und Wirtschaftskrise bereits knapp nach ihrem Ausbruch in den öffentlichen Fokus. Bei Vermögen zeigen sich die Verluste zunächst in den Unternehmenswerten. Auch wenn diese unterschiedlich betroffen sind (Häring, 2020), reagieren Bewertungen auf den Finanzmärkten höchst volatil auf Abschwünge. Auch in der ersten Phase der Finanz- und Wirtschaftskrise nach 2008 konzentrierte sich die Wirtschaftspresse zunächst auf die Topvermögen, allerdings sind im Fall der Gesundheits- und Wirtschaftskrise die Auswirkungen auf die breite Mehrheit der Bevölkerung, die geringe bis mittlere Vermögen besitzen und deren Einkommen hauptsächlich durch Löhne und Selbständigen-Einkommen und nicht durch Vermögenseinkommen bestimmt sind, bereits in diesem Frühstadium klarer absehbar.

In Deutschland stagnierte und fiel das Nettovermögen nach der Finanz- und Wirtschaftskrise im Jahr 2010 und stieg davor und danach stark an (Albers et al., 2020). Die Ungleichheit sank von 2008 auf 2013, und stagniert seither (gemessen mit dem Gini-Koeffizienten) bzw. hat das Vorkrisenniveau wieder erreicht (gemessen mit dem Anteil des Top 1 %). In den USA dagegen stieg die Ungleichheit gemessen am Verhältnis des Durchschnitts- zum Medianvermögen wegen der hohen Hypothekenverschuldung der mittleren Haushalte in der Krise stark an (Wolff, 2017); der Anteil des reichsten 1 % am Nettovermögen stagnierte zwischen 2007 und 2010 bei etwa 30 %, um dann bis 2016 auf 35 % anzusteigen (Bricker und Henriques Volz, 2020).

Das Vermögen der oberen Vermögensgruppen ist somit in den USA nach der Krise deutlich stärker gewachsen, und auch im Euroraum zeigt der HFCS seit seiner ersten Welle 2010 trotz der Wachstumsschwäche keinen anhaltenden Rückgang der Vermögensungleichheit. Langfristige Modelle unterstreichen diese Entwicklung sowohl aus theoretischer als auch aus empirischer Perspektive. In einem analytischen makroökonomischen Wachstumsmodell zeigen wir (Ederer und Rehm, 2020b), dass die langfristige Entwicklung der Vermögensverteilung mitnichten zwingend dazu führt, dass sich das gesamte Vermögen bei den Reichen konzentrieren wird, wie Pikettys (2017) $r > g$ (Kapitalrenditen übersteigen die Wachstumsrate) implizieren würde: ArbeitnehmerInnen besitzen auch in der langen Frist einen positiven, stabilen Anteil am gesamten Privatvermögen. Allerdings prognostiziert das Modell, das wir für zehn europäische Länder kalibrieren, in allen Fällen einen weiteren Anstieg der Vermögensungleichheit.

In Simulationen stellen wir zudem fest, dass Pikettys empirische „stilisierten Fakten" die Übergangsfrist zum langfristigen Gleichgewicht für Europa treffend charakterisieren (Ederer und Rehm, 2020a): Das Kapital-Einkommensverhältnis steigt, die Vermögens- und Einkommensungleichheit nimmt ebenfalls zu, und das Wirtschaftswachstum schwächt sich ab. Erst in der sehr langen Frist stabilisieren sich diese Werte auf hohem Niveau, ebenso wie das Verhältnis von Kapitaleinkommen und Wachstumsrate. Eine Vermögensteuer kann dagegen diesen Anstieg der Vermögensungleichheit neutralisieren.

Das unumgängliche „Schockgefrieren" der Wirtschaft in der derzeitigen Krisensituation verursacht hohe Staatsausgaben. Eine Vermögensteuer oder -abgabe könnte aufgrund der in diesem Beitrag dokumentierten hohen Ungleichheit bei Vermögen in Deutschland trotz hoher Freibeträge bei sehr guter Treffsicherheit, was die ökonomische Leistungsfähigkeit angeht, ein substanzielles Aufkommen generieren. Ein konkreter Vorschlag für die Umsetzung einer solchen Vermögenssteuer wird etwa in Bach und Thiemann (2016) entwickelt. Eine vergleichbare Wirkung hätte aufgrund der üblichen Stundung eine einmalige Vermögensabgabe (Bach, 2012), zugleich bedarf es einer vorsichtigen Abwägung mit alternativen Instrumenten wie einer Erbschaftsteuer (Bach, 2016). Landeis et al. (2020) schlagen eine zeitlich begrenzte Vermögensteuer auf EU-Ebene zum Abbau der Krisenkosten vor.

Aus haushaltspolitischer Sicht sollte eine Vermögenssteuer erst nach der Krise eingeführt, die Diskussion um eine gerechte Verteilung der Krisenfinanzierung sollte jedoch nicht auf die lange Bank geschoben werden. Nach der Finanz- und Wirtschaftskrise 2008 war eine unkoordinierte und unangemessene Reaktion sowohl der Geld- als auch der Fiskalpolitik mit für den „double dip" des Wachstums im Euroraum verantwortlich, während die USA im gleichen Zeitraum langsam aus der Krise wuchsen. Diesen Fehler gilt es nach der Gesundheits- und Wirtschaftskrise 2020 nicht zu wiederholen. Die sehr ungleiche Verteilung der Vermögen bietet einen möglichen Ansatzpunkt dafür.



Literatur

Albers T Bartels C Schularick M The Distribution of Wealth in Germany, 1895–2018 ECONtribute Working Paper 2020
Bach S Vermögensabgaben–ein Beitrag zur Sanierung der Staatsfi nanzen in Europa DIW Wochenbericht 2012 28 3 11
Bach S Erbschaftsteuer, Vermögensteuer oder Kapitaleinkommensteuer: Wie sollen hohe Vermögen stärker besteuert werden? DIW Discussion Papers 2016 1619
Bach S Thiemann A Hohes Aufkommenspotential bei Wiedererhebung der Vermögensteuer DIW Wochenbericht 2016 4 79 89
Bardt H Dullien S Hüther M Rietzler K Für eine solide Finanzpolitik: Investitionen ermöglichen! IW/IMK Report 2019 152
Bricker J Henriques Volz A Wealth concentration levels and growth: 1989-2016 FEDS Notes 2020
Deutsche Bundesbank Vermögen und Finanzen privater Haushalte in Deutschland: Ergebnisse der Vermögensbefragung 2017 Monatsbericht 2019 13
Ederer S Rehm M Making Sense of Piketty’s ‘Fundamental Laws’ in a Post-Keynesian Framework: The Transitional Dynamics of Wealth Inequality Review of Keynesian Economics 2020 8 2 195 219 10.4337/roke.2020.02.04
Ederer S Rehm M Will Wealth Become More Concentrated in Europe? Evidence from a Calibrated Post-Keynesian Model Cambridge Journal of Economics 2020 44 1 55 72
European Commission Eurostat data base 2020
Europäische Zentralbank The Household Finance and Consumption Survey: Methodological report for the 2017 wave, Household Finance and Consumption Network Statistics Paper Series 2020
Fessler P Lindner P Schürz M Eurosystem Household Finance and Consumption Survey 2017 First results for Austria, Oesterreichische Nationalbank 2019
Fessler P Schürz M The functions of wealth: renters, owners and capitalists across Europe and the United States Oesterreichische Nationalbank, Working Papers 2018 223
Frick J Grabka M Vermögen in Deutschland wesentlich ungleicher verteilt als Einkommen DIW Wochenbericht 2007 45
Häring N Weshalb einige Superreiche nicht ganz so viel leiden Handelsblatt 2020 26
Internationaler Währungsfonds IWF Estimating the stock of public capital in 170 countries 2017
Kranawetter P Rehm M Selbsteinschätzung beim Vermögen A&W Blog 2018 12
Landeis C Saez E Zucman G A progressive European wealth tax to fund the European COVID response Vox Blog 2020
Mazzucato M The Entrepreneurial State 2014
Oesterreichische Nationalbank OeNB Dashboard 3 2020
Piketty T Capital in the 21st Century 2014
Rinke A Fünf Lehren aus Wuhan–„Sechs-Stunden-Schichten retten Leben“ Reuters 2020 31
Schneebaum A Mader K Rehm M Hollan K The Gender Wealth Gap across European Countries Review of Income and Wealth 2018 64 2 295 331 10.1111/roiw.12281
Sierminska E Frick J Grabka M Examining the gender wealth gap Oxford Economic Papers 2010 62 4 669 690 10.1093/oep/gpq007
Sierminska E Piazzalunga D Grabka M Transitioning towards more equality? Wealth gender differences and the changing role of explanatory factors over time LISER Working Paper Series 2018
Stiglitz J Fitoussi J-P Durand M Beyond GDP. Measuring what counts for economic and social performance OECD Publishing 2018
Wolff E Household wealth trends in the United States, 1962 to 2016: Has middle class wealth recovered? NBER Working Paper 2017
